# Maternal Characteristics Associated With Congenital Anomalies: An Exploratory Case Series in a Secondary Care Hospital

**DOI:** 10.7759/cureus.108650

**Published:** 2026-05-11

**Authors:** Daniela Juárez-Melchor, Pablo Omar Rodríguez-Hurtado, Aurea Vera-Loaiza, Alan Alberto Pérez-Arzola, Yazmin Hernández-Castañeda, Oscar Olivares-Huerta, Jonathan Cervantes-Larios, Tania Alejandra Guzmán-Santiago, Wilbert Salazar-Bonilla, Israel Enrique Crisanto-López

**Affiliations:** 1 Center for Health Sciences, University of Guadalajara, Guadalajara, MEX; 2 Department of Medical Genetics, General Hospital Zone No. 20, Mexican Social Security Institute, Puebla, MEX; 3 Department of Health Sciences, Universidad de las Américas Puebla, Puebla, MEX; 4 Department of Neurological Genetics, National Institute of Neurology and Neurosurgery, Mexico City, MEX; 5 Department of Dermatological Genetics, General Hospital of Mexico, Mexico City, MEX; 6 Department of Family Medicine, Family Medicine Unit No. 6, Mexican Social Security Institute, Puebla, MEX; 7 Reproductive Biology Laboratory, Mexican Social Security Institute, Puebla, MEX; 8 Department of Medicine, Meritorious Autonomous University of Puebla, Puebla, MEX

**Keywords:** cleft lip & palate, cleft lip/palate, congenital abnormalities, folic acid supplementation, non-syndromic patients, prenatal care access

## Abstract

Background

Congenital anomalies are a public health concern and the leading cause of infant mortality, particularly in low- and middle-income countries. Established risk factors include pregestational diabetes, maternal obesity, folic acid deficiency, and limited access to prenatal care. The aim of this exploratory study was to describe maternal characteristics in relation to isolated and multiple non-syndromic congenital anomalies in a series of cases from a secondary-care hospital in Puebla, Mexico.

Materials and methods

An observational, cross-sectional, exploratory case series with ambispective data collection was conducted in the Genetics Department of General Hospital Zone No. 20 of the Mexican Social Security Institute in Puebla, Mexico. The study included 31 mothers of patients with non-syndromic congenital anomalies. Data collected included sociodemographic characteristics, anthropometric measurements, and environmental exposures. Congenital anomalies were categorized as isolated or multiple. Categorical variables were compared using Fisher's exact test and continuous variables using the Mann-Whitney U test.

Results

A total of 31 mothers of patients with congenital anomalies were analyzed. Twenty-one (67.7%) cases presented isolated anomalies, and 10 (32.3%) multiple anomalies, with craniofacial anomalies being the most frequent. The mean maternal age at the time of pregnancy was 27.71 ± 5.58 years. Higher frequencies were observed among mothers with higher educational attainment, employment, and exposure to teratogens; however, these differences did not reach statistical significance.

Conclusion

In this exploratory case series, no statistically significant differences were found between maternal characteristics and the presence of isolated or multiple congenital anomalies. Observed differences should be interpreted in the context of the study design and limited sample size, and these findings are hypothesis-generating and require confirmation in larger, controlled studies.

## Introduction

Congenital anomalies are structural or functional defects occurring during intrauterine life and can be identified prenatally, at birth, or later in life [1]. Congenital anomalies can be classified based on the number and relatedness of defects present in isolated or multiple. Individual congenital anomalies are categorized by their pathogenetic mechanism: malformation, deformation, disruption, and dysplasia. In this way, an isolated anomaly is a single structural defect occurring alone, or a defect accompanied only by secondary anomalies that are developmentally related to the primary defect. Multiple congenital anomalies are two or more apparently unrelated major anomalies affecting different organ systems [2,3].

When multiple anomalies co-occur, they are further classified by the nature of their relationship, such as syndrome, sequence, association, and developmental field defect. Based on whether a recognized pattern or underlying cause links the defects in syndromic cases, the anomaly occurs as part of a recognized pattern of malformations with a known or presumed common etiology (chromosomal syndromes, genomic syndromes, monogenic syndromes), and in non-syndromic cases, the anomaly occurs without a recognized underlying syndrome or pattern and is generally considered etiologically distinct from syndromic cases [3].

Congenital anomalies represent a major public health concern. Infant mortality has been increasing over the years, from 4.6% in 2000 to 7.6% in 2019. About 94% of the affected neonates are from low- and middle-income countries, where mortality rates are more than double those in high-income countries. Congenital anomalies have been reported to affect about 6% of live births worldwide (~7.9 million per year). These anomalies are the fourth leading cause of death in children under five years of age, accounting for 9.4% of all child deaths [4,5].

Mexico has a large burden of congenital anomalies. The Epidemiological Surveillance System for Birth Defects (SVEDAN) reported 192,273 deaths due to congenital anomalies from 2000 [6]. The second quarter report of 2025 identified 1,528 cases of congenital anomalies with an incidence of 145.6 cases per 100,000 live births [7].

Congenital anomalies can be prevented through screening, taking in adequate nutrients, avoiding harmful substances, and prenatal care [5]. Maternal risk factors include pregestational diabetes and placental complications or hemorrhage, followed by maternal obesity, advanced maternal age, hypertension, folic acid deficiency, exposure to teratogenic medications, ionizing radiation, alcohol use, and smoking [8,9]. It has been reported that a lack of prenatal care is associated with an increased risk of a congenital anomaly [10].

Therefore, the aim of this exploratory study was to describe maternal characteristics in relation to isolated and multiple non-syndromic congenital anomalies in a series of cases from a secondary-care hospital in Puebla, Mexico.

## Materials and methods

Study design and setting

An observational, cross-sectional, exploratory case series with ambispective data collection was conducted at General Hospital Zone No. 20, Mexican Social Security Institute, a secondary-level facility in Puebla, Mexico. The study was reviewed by the local ethics and research committees, with subsequent approval and registration number R-2023-2108-127.

Patient selection and study population

A targeted search was conducted in the consultation records of the Medical Genetics Service, where clinical geneticists evaluate patients. Records from patients assessed between 2023 and 2025 were screened.

Congenital anomalies were assessed by geneticists according to a hierarchical classification system, as recommended by the European Surveillance of Congenital Anomalies (EUROCAT) guidelines. This system integrates the following dimensions: chromosomal syndromes and monogenic syndromes take precedence, followed by teratogenic syndromes, other syndromic associations, multiple unrelated anomalies, anomalies within the same organ system, and finally isolated single anomalies. Thus, these are defined either syndromic with a common underlying etiology (including chromosomal abnormalities, copy number variants, or monogenic disorders) or non-syndromic, defined as anomalies with a likely multifactorial origin.

The inclusion criteria are the following: patients with clinically confirmed non-syndromic congenital anomalies and availability of maternal information. The exclusion criteria are the following: patients with confirmed or suspected syndromic diagnoses, incomplete clinical records, or inability to contact the mother. A total of 585 records were reviewed; 554 were excluded based on these criteria, and the final analytic sample included 31 cases.

**Figure 1:**
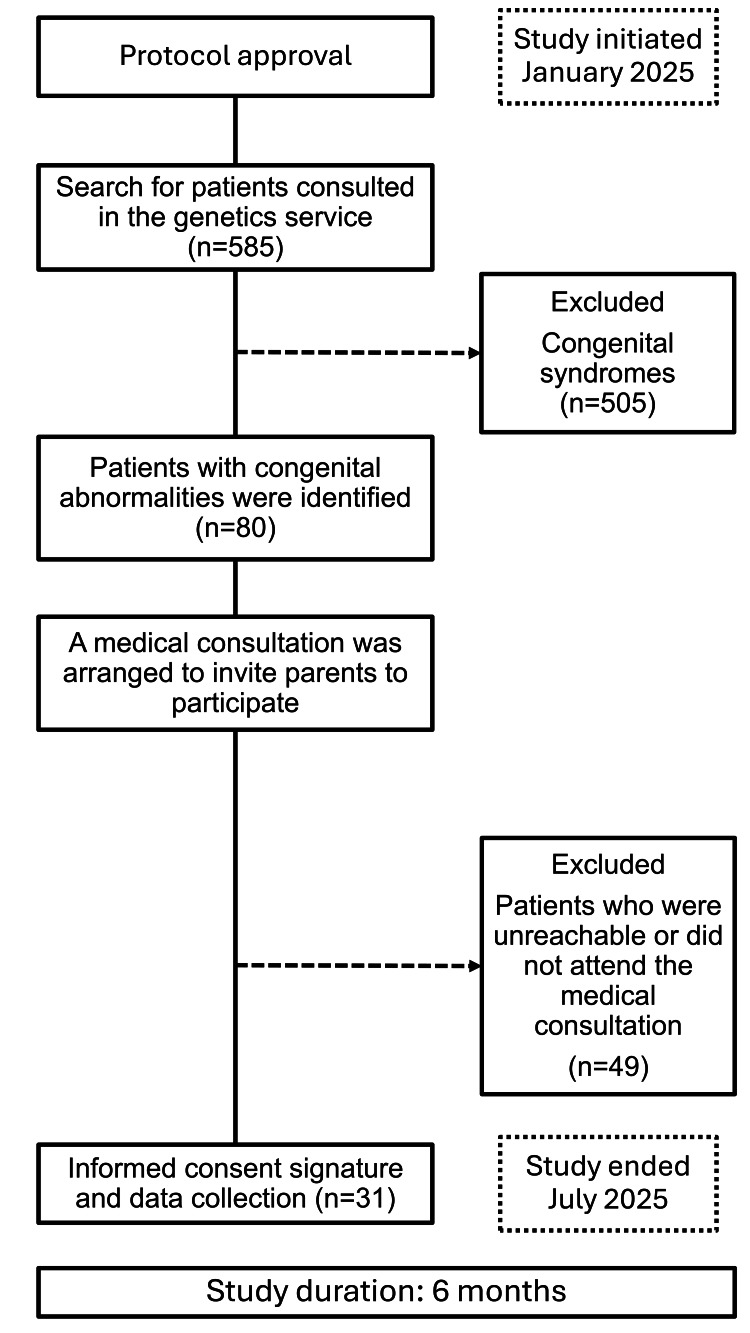
Participant flowchart

The study population consisted of biological mothers of patients with non-syndromic congenital anomalies who agreed to participate and provided written informed consent. A consecutive non-probabilistic sampling method was used. The study duration was six months (Figure 1).

Medical history

Maternal data were collected from medical records and complemented with maternal self-reports during a structured clinical interview. The variables collected included maternal age at pregnancy, gestational age at diagnosis, anthropometric measurements (height and weight) used to calculate body mass index (BMI), occupation, education level, alcohol and tobacco use, exposure to substances, folic acid intake, teratogen exposure, perigestational infections, history of gestational diabetes, hypertensive disorders of pregnancy, and fever during pregnancy.

Definition of exposure variables

Folic acid use was defined as maternal supplementation before or during the first trimester of pregnancy.

Teratogen exposure was defined as self-reported exposure to substances with known or suspected teratogenic potential, including solvents, dyes, and wood smoke, during pregnancy.

Perigestational infections were defined as maternal infections that occurred during pregnancy or the periconceptional period as determined by clinical diagnosis or maternal report.

Fever was defined as maternal report or documented temperature ≥38°C during pregnancy.

Classification of congenital anomalies

Congenital anomalies were classified as isolated (single anomaly affecting one organ system) or multiple (two or more anomalies involving different organ systems). Additionally, anomalies were categorized by the affected system based on SVEDAN classification: central nervous system, craniofacial, cardiovascular, gastrointestinal, genitourinary, and limb anomalies.

Statistical analysis

Descriptive statistics were calculated for all study variables. The Shapiro-Wilk test was used to assess normality. The variables with normal distribution were expressed as mean and standard deviation, and the non-normally distributed variables were reported as median and interquartile range (IQR). Categorical variables were summarized using frequencies and percentages.

Comparisons between isolated and multiple congenital anomalies were performed using Fisher's exact tests for categorical variables and the Mann-Whitney U tests for continuous variables, due to the small sample size. All analyses were performed using Statistical Product and Service Solutions (SPSS, version 25; IBM SPSS Statistics for Windows, Armonk, NY).

## Results

Data were collected from patients who attended the medical genetics service. A total of 585 patients were identified, of whom eighty had non-syndromic congenital anomalies. Subsequently, 31 mothers who could be contacted were invited to participate; appointments were scheduled to provide study information and obtain written informed consent. The characteristics of the congenital anomalies found are shown in Table 1.

**Table 1:** Characteristics of the congenital anomalies reported in the patients CNS: Central nervous system

Anomaly presentation	Frequency (n=31)	Percentage
Isolated	21	67.7%
Multiple	10	32.3%
Classification of congenital anomalies (isolated), (n=21)		
CNS	4	19.05%
Craniofacial	13	61.90%
Cardiovascular	1	4.76%
Gastrointestinal	2	9.53%
Genitourinary	0	-
Limbs	1	4.76%

Maternal characteristics of children with non-syndromic congenital anomalies

The mean maternal age at the time of consultation was 28.97 ± 5.13 years, and the mean maternal age at the time of pregnancy was 27.71 ± 5.58 years. The median gestational age at diagnosis was six weeks (IQR: 8, range: 3-27). Maternal characteristics are reported in Table 2. The mean maternal BMI was 25.13 ± 5.51 kg/m^2^.

**Table 2:** Characteristics of the mothers of patients with congenital anomalies ^a^The teratogens to which participants were exposed included solvents, dyes, wood smoke, compressed air, textile fibers, and X-ray exposure during the critical period of embryogenesis. ^b^Perigestational infections included 26 cases of urinary tract infections during the first trimester, one confirmed dengue virus infection at 22 weeks of gestation, and one case of pharyngotonsillitis.

Occupation	Frequency (n=31)	Percentage
Homemakers	20	64.5%
Paid employment	11	35.5%
Education		
Basic education	11	35.5%
Upper secondary education or higher	20	64.5%
Substances abuse (alcohol)		
Yes	4	12.9%
No	27	87.1%
Gestational age (weeks) at folic acid initiation		
Before 10 weeks	19	61.3%
After 10 weeks	12	38.7%
Exposures during pregnancy		
Teratogens^a^	6	19.4%
Perigestational infections^b^	26	83.9%
COVID-19 vaccination	2	6.5%
Fever	5	16.1%
Body mass index		
Normal weight	17	54.8%
Overweight	7	22.6%
Obesity I	6	19.4%
Obesity II	-	-
Obesity III	1	3.2%

A bivariate analysis was performed using Fisher's exact test and the Mann-Whitney U test (Table 3). No statistically significant differences were identified between maternal characteristics and the presence of isolated or multiple congenital anomalies. A higher frequency of multiple congenital anomalies was observed among offspring of mothers with paid employment (54.5%) compared to homemakers (20%) (p=0.106). Similarly, mothers with a high school education or higher had a higher frequency of multiple anomalies (45%) compared to those with a basic education (9.1%) (p=0.055). A higher proportion of multiple congenital anomalies was also observed among mothers exposed to teratogens (50%) compared to non-exposed mothers (28%) (p=0.358). These findings are descriptive and should be interpreted with caution.

**Table 3:** Bivariate analysis of maternal factors associated with congenital anomalies Values are expressed as n (%) or median (interquartile range). Fisher's exact test was used for categorical variables and the Mann-Whitney U test for continuous variables; statistical significance was set at p < 0.05. OR: Odds ratio; CI: Confidence interval; IQR: Interquartile range

Variables		Congenital anomalies, Isolated, n (%)	Congenital anomalies, Multiple, n (%)	OR (CI 95%)	p
Occupation	Homemakers	16 (80%)	4 (20%)	4.8 (0.95-24.14)	0.106
	Paid employment	5 (45.5%)	6 (54.5%)		
Education	Basic education	10 (90.9%)	1 (9.1%)	8.18 (0.87-76.58)	0.055
	Upper secondary education or higher	11 (55%)	9 (45%)		
Maternal substance abuse	Si	3 (75%)	1 (25%)	1.5 (0.13-16.54)	1.000
	No	18 (66.7%)	9 (33.3%)		
Gestational age (weeks) at folic acid initiation	Before 10 weeks	14 (73.7%)	5 (26.3%)	2.000 (0.43-9.29)	0.447
	After 10 weeks	7 (58.3%)	5 (41.7%)		
Exposure to teratogens	Yes	3 (50%)	3 (50%)	2.570 (0.41-15.87)	0.358
	No	18 (72%)	7 (28%)		
Perigestacional infection	Yes	18 (69.2%)	8 (30.8%)	0.666 (0.09-4.80)	1.000
	No	3 (60%)	2 (40%)		
COVID-19 vaccination	Yes	1 (50%)	1 (50%)	0.450 (0.02-8.02)	1.000
	No	20 (69%)	9 (31%)		
Fever	Yes	5 (100%)	0 (-)	-	0.147
	No	16 (61.5%)	10 (38.5%)		
Maternal BMI >25	<25 kg/m^2^	12 (70.6%)	5 (29.4%)	0.750 (0.17-3.40)	1.000
	>25 kg/m^2^	9 (64.3%)	5 (35.7%)		
Maternal age at pregnancy median (IQR)		27 (25-29)	29.5 (25-33)	-	0.566
Maternal BMI, median (IQR)		24 (21.3-26.9)	25.5 (21.1-30.8)	-	0.574

## Discussion

In this exploratory case series, no statistically significant differences were found between the maternal variables assessed and the presence of isolated or multiple congenital anomalies. However, a higher proportion was observed in relation to maternal variables, such as educational attainment, occupation, and exposure to teratogens. These observations should be interpreted in the context of the exploratory design and small sample size.

A higher frequency of multiple congenital anomalies was observed among children of mothers with a high school education or higher. Previous studies have shown that lower maternal education is associated with an increased risk of congenital anomalies, while high educational attainment has been described as a protective factor against pregnancy outcomes [11-13]. These differences observed in the present study may reflect variations in access to healthcare services and tools for prenatal diagnosis that may influence the likelihood of detection of congenital anomalies.

Similarly, a higher frequency of multiple anomalies was observed among employed mothers, although these differences were not statistically significant. Prior studies have suggested that certain maternal occupations, such as exposure to chemical or manual labor, may be associated with an increased risk of congenital anomalies [14,15].

A higher proportion of multiple anomalies was observed in mothers exposed to teratogens. Although this difference was not statistically significant, current evidence supports the biological plausibility that exposure to teratogenic agents during critical periods of development may increase the risk of anomalies involving multiple organ systems [16]. For example, maternal exposure to pesticides during the periconceptional period has been associated with a 2.39-fold increased risk of congenital defects and a 3.14-fold increased risk of cardiovascular defects [17]. Furthermore, prenatal exposure to air pollutants has been related to defects: particulate matter has been related to genitourinary defects, neural tube defects, and overall birth defects [18,19].

No difference was found between isolated and multiple anomalies in this sample in relation to perigestational infection and maternal age. However, in previous studies, these factors have been related to increased risk of congenital anomalies [4,8,9,20]. In this way, first-trimester infections have been associated with a 63% increased risk of congenital heart defects [21]. Severe acute respiratory infections during the first trimester have been linked to a 3.64-fold increased risk of major cardiovascular anomalies, and periconceptional genitourinary infections (from three months before to three months after conception) have been associated with an increased risk of gastroschisis [22].

Women of advanced maternal age have a higher risk of having any congenital anomaly (OR: 1.64) [23]. Similarly, young maternal age is associated with an increased risk of any non-chromosomal anomaly, including anomalies of the digestive system and musculoskeletal system. This phenomenon follows a U-shaped pattern, in which both noticeably young and advanced maternal age are associated with diverse types of non-chromosomal anomalies [24].

Concerning folic acid intake, no differences were observed between groups according to the timing of supplementation. Current evidence supports a protective effect of folic acid against neural tube defects, particularly when supplementation occurs during the periconceptional period [25,26].

Maternal BMI was similar between the groups with isolated and multiple congenital anomalies. Although overweight and obesity have been associated with an increased risk of congenital anomalies overall, no differences were observed between groups in this study, and the anomalies appeared to be independent of whether these were classified as isolated or multiple [4,8,9]. In addition, pre-pregnancy obesity (BMI ≥ 30 kg/m²) increases the risk of birth defects by 21-38%, especially malformations of the circulatory system, neural tube defects, cleft palate, hydrocephalus, and limb reduction anomalies. Maternal overweight (BMI 25-29.9) also increases the risk. Obesity may reduce folate levels and diminish the protective effect of folic acid against neural tube defects. Furthermore, when obesity is combined with gestational diabetes, an additive effect on the risk of cardiac malformations has been reported [27,28].

This study should be interpreted as a small exploratory case series, and the findings are hypothesis-generating and do not allow the identification of risk factors or causal relationships. There are important limitations. First, the small sample size resulted in low statistical power and wide confidence intervals. Second, the lack of a control group of mothers with healthy children prevents the determination of whether the observed maternal characteristics are risk factors or simply common in this population. The sampling approach has the potential for selection and detection bias. Larger, controlled studies are required to confirm or refute these observations.

Public health implications discussed are supported by existing literature rather than findings of this study alone. Preventive strategies such as folic acid supplementation and reduction of exposure to teratogens remain essential and are supported by prior evidence.

## Conclusions

No statistically significant difference was observed between maternal characteristics and the presence of isolated or multiple congenital anomalies in this exploratory case series study. However, higher frequencies were observed in mothers with higher education, employed, and teratogen exposure, which should be interpreted in view of the study design and small sample size. These findings are hypothesis-generating and cannot identify risk factors or causal relationships due to the lack of a control group and small sample size. Larger, controlled studies are required to confirm or refute these observations.

## References

[1] (2026). World Health Organization: congenital disorders. https://www.who.int/news-room/fact-sheets/detail/birth-defects.

[2] Tonkin MA (2017). Classification of congenital anomalies of the hand and upper limb. J Hand Surg Eur Vol.

[3] Benjamin RH, Nguyen JM, Drummond-Borg M (2024). Classification of isolated versus multiple birth defects: an automated process for population-based registries. Am J Med Genet A.

[4] Perin J, Mai CT, De Costa A (2023). Systematic estimates of the global, regional and national under-5 mortality burden attributable to birth defects in 2000-2019: a summary of findings from the 2020 WHO estimates. BMJ Open.

[5] Kang L, Cao G, Jing W, Liu J, Liu M (2023). Global, regional, and national incidence and mortality of congenital birth defects from 1990 to 2019. Eur J Pediatr.

[6] (2026). Ministry of Health. General Directorate of Epidemiology: standardized procedures for the epidemiological surveillance of birth defects 2023 [Article in Spanish]. https://www.gob.mx/salud/documentos/defectos-al-nacimiento-2023.

[7] (2025). Quarterly report epidemiological surveillance system for birth defects [Article in Spanish]. https://www.gob.mx/cms/uploads/attachment/file/1010286/InformeTrimestral_SVEDAN_2doTrim_2025.pdf.

[8] Luke B, Fisher SC, Forestieri NE (2025). Maternal, reproductive and perinatal factors and the risks of birth defects: traditional and emerging factors. Reprod Biomed Online.

[9] Lee KS, Choi YJ, Cho J (2021). Environmental and genetic risk factors of congenital anomalies: an umbrella review of systematic reviews and meta-analyses. J Korean Med Sci.

[10] Fernandes QH, Paixão ES, da Conceição Nascimento Costa M, Teixeira MG, Barreto ML, Acosta AX (2025). Maternal and gestational factors associated with congenital anomalies among live births: a nationwide population-based study in Brazil from 2012 to 2020. BMC Pregnancy Childbirth.

[11] Trevilato GC, Riquinho DL, Mesquita MO, Rosset I, Augusto LG, Nunes LN (2022). Congenital anomalies from the perspective of social determinants of health [Article in Spanish]. Cad Saude Publica.

[12] Rogne T, Gill D, Liew Z, Shi X, Stensrud VH, Nilsen TI, Burgess S (2024). Mediating factors in the association of maternal educational level with pregnancy outcomes: a Mendelian randomization study. JAMA Netw Open.

[13] Swaminathan A, Lahaie Luna M, Rennicks White R (2022). The influence of maternal and paternal education on birth outcomes: an analysis of the Ottawa and Kingston (OaK) birth cohort. J Matern Fetal Neonatal Med.

[14] Yang Y, Herdt ML, Hosler AS, Desrosiers TA, Howley MM (2025). Association between maternal occupation as a cleaner/maid/janitor during early pregnancy and selected birth defects in the National Birth Defects Prevention Study. Occup Environ Med.

[15] Siegel MR, Rocheleau CM, Broadwater K (2022). Maternal occupation as a nail technician or hairdresser during pregnancy and birth defects, National Birth Defects Prevention Study, 1997-2011. Occup Environ Med.

[16] Zhang H, Li Y, Zhang X (2022). Potential occupational exposure of parents to endocrine disrupting chemicals, adverse birth outcomes, and the modification effects of multi-vitamins supplement and infant sex. Ecotoxicol Environ Saf.

[17] Liu F, Li X, Chen J, Huang Y, Dang S (2024). Maternal pesticide exposure and risk of birth defects: a population-based cross-sectional study in China. Front Public Health.

[18] Al Noaimi G, Yunis K, El Asmar K (2021). Prenatal exposure to criteria air pollutants and associations with congenital anomalies: a Lebanese national study. Environ Pollut.

[19] Sun S, Zhang Q, Sui X (2021). Associations between air pollution exposure and birth defects: a time series analysis. Environ Geochem Health.

[20] Morris JK, Bergman JE, Barisic I (2024). Surveillance of multiple congenital anomalies; searching for new associations. Eur J Hum Genet.

[21] Su H, Guo E, Woodward M (2026). First trimester maternal infections and offspring congenital heart defects: a meta-analysis. Eur Heart J.

[22] Feldkamp ML, Arnold KE, Krikov S, Reefhuis J, Almli LM, Moore CA, Botto LD (2019). Risk of gastroschisis with maternal genitourinary infections: the US National birth defects prevention study 1997-2011. BMJ Open.

[23] Ahn D, Kim J, Kang J, Kim YH, Kim K (2022). Congenital anomalies and maternal age: a systematic review and meta-analysis of observational studies. Acta Obstet Gynecol Scand.

[24] Pethő B, Mátrai Á, Agócs G (2023). Maternal age is highly associated with non-chromosomal congenital anomalies: analysis of a population-based case-control database. BJOG.

[25] Barry MJ, Nicholson WK, Silverstein M (2023). Folic acid supplementation to prevent neural tube defects: US Preventive Services Task Force reaffirmation recommendation statement. JAMA.

[26] Iskandar BJ, Finnell RH (2022). Spina bifida. N Engl J Med.

[27] Liu W, Ren L, Fang F, Chen R (2024). Maternal pre-pregnancy overweight or obesity and risk of birth defects in offspring: population-based cohort study. Acta Obstet Gynecol Scand.

[28] Hedermann G, Hedley PL, Thagaard IN, Krebs L, Ekelund CK, Sørensen TI, Christiansen M (2021). Maternal obesity and metabolic disorders associate with congenital heart defects in the offspring: a systematic review. PLoS One.

